# Evaluation of the long-term efficacy and safety of an imidacloprid 10%/flumethrin 4.5% polymer matrix collar (Seresto^®^) in dogs and cats naturally infested with fleas and/or ticks in multicentre clinical field studies in Europe

**DOI:** 10.1186/1756-3305-5-66

**Published:** 2012-03-31

**Authors:** Dorothee Stanneck, Julia Rass, Isabel Radeloff, Eva Kruedewagen, Christophe Le Sueur, Klaus Hellmann, Klemens Krieger

**Affiliations:** 1 Bayer Animal Health GmbH (BAH), Monheim, Germany; 2 KLIFOVET AG, Munich, Germany; 3 Bayer Santé S.A.S, Puteaux, France

**Keywords:** *Ctenocephalides felis*, *Ctenocephalides canis*, *Archaeopsylla erinacei*, *Pulex irritans*, *Dermacentor reticulatus*, *Ixodes hexagonus*, *Ixodes ricinus*, Fleas, Ticks, Efficacy, Safety, Imidacloprid, Flumethrin, Collar, Field

## Abstract

**Background:**

The objective of these two GCP multicentre European clinical field studies was to evaluate the long-term efficacy and safety of a new imidacloprid/flumethrin collar (Seresto^®^, Bayer AnimalHealth, Investigational Veterinary Product(IVP)) in dogs and cats naturally infested with fleas and/or ticks in comparison to a dimpylat collar ("Ungezieferband fuer Hunde/fuer Katzen", Beaphar, Control Product (CP)).

**Methods:**

232 (IVP) and 81 (CP) cats and 271(IVP) and 129 (CP) dogs were treated with either product according to label claims and formed the safety population. Flea and tick counts were conducted in monthly intervals for up to 8 months in the efficacy subpopulation consisting of 118 (IVP) + 47 (CP) cats and 197 (IVP) + 94 (CP) dogs. Efficacy was calculated as reduction of infestation rate within the same treatment group and statistically compared between the two treatment groups.

**Results:**

Preventive efficacy against fleas in cats/dogs varied in the IVP group between 97.4%/94.1% and 100%/100% (overall mean: 98.3%/96.7%) throughout the 8 month period and in the CP group between 57.1%/28.2% and 96.1%/67.8% (overall mean: 79.3%/57.9%). Preventive efficacy against ticks in cats/dogs varied in the IVP group between 94.0%/91.2% and 100%/100% (overall mean: 98.4%/94.7%) throughout the 8 month period and in the CP group between 90.7%/79.9% and 100%/88.0% (overall mean: 96.9%/85.6%). The IVP group was statistically non-inferior to the CP group, and on various assessment days, statistical superiority was proven for flea and tick count reduction in dogs and cats. Both treatments proved to be safe in dogs and cats with mainly minor local observations at the application site. There was moreover, no incidence of any mechanical problem with the collar in dogs and cats during the entire study period.

**Conclusions:**

The imidacloprid/flumethrin collar proved to reduce tick counts by at least 90% and flea counts by at least 95% for a period of at least 7-8 months in cats and dogs under field conditions. Therefore, it can be used as sustainable long-term preventative, covering the whole flea and tick season.

## Background

Fleas and ticks are common ectoparasites in dogs and cats and are present in many areas of the world, in differing intensity depending on the climatic conditions. Fleas are present in all areas of Europe, with *Ctenocephalides felis *being the most frequent flea species on our companion animals followed by *Ctenocephalides canis*. The main European tick species are *Rhipicephalus sanguineus *(dog); *Ixodes ricinus *(cat and dog), *Dermacentor reticulatus *(dog) and *Rhipicephalus turanicus *(cat). The latter species is not very well recognized but recently acknowledged to be the species present on cats instead of the nearly identical but very dog specific *R. sanguineus *[1]. Fleas tend to occur from spring to winter and are capable of acting as vectors for several diseases, e.g. bartonellosis and tapeworms, and can cause flea allergic dermatitis (FAD). Ticks peak from early spring to late autumn and are important vectors for several diseases, e.g. borreliosis, anaplasmosis, ehrlichiosis and babesiosis. In humans they can transmit tick encephalitis, borreliosis and anaplasmosis. Both fleas and ticks play an important role as vectors [2]. Therefore, effective measures against these parasites are important in preventing feline, canine and human disease [3].

While there are many authorized products containing different active ingredients for the prevention of fleas, there are only a few products available against ticks. Products containing fipronil are almost the only option for tick treatment of cats and dogs as the other common acaricides, permethrin and amitraz, are indicated for dogs only [4,5] and are contraindicated for use in cats due to serious safety concerns. Flumethrin, the acaricidal active component in Kiltix^® ^collars (propoxur 10%/flumethrin 2.25%), is a highly potent acaricide known to be safe in various animal species including cattle, sheep and dogs but also cats: the Kiltix^® ^collar was, up to-date the only broadly marketed pyrethroid containing formulation suitable for cats.

Formulations of these active ingredients are generally designed for topical application with the most common versions being collars and low volume fluids (spot ons): Collars have been used frequently for the treatment and prevention of flea and tick infestations in dogs and cats in the past decades in Europe and abroad [6,7]. However, with the launch of spot-on topical formulations and concerns about the perceived potential risk for free roaming cats to be trapped by any salient piece of wood or other rigid material, collars have been less involved in the growing market for flea and tick control.

Seresto^® ^(Bayer Animal Health), a new collar for dogs and cats, provides long term broad spectrum parasiticidal activity by combining the insecticidal properties of imidacloprid with the acaricidal properties of flumethrin. The collar matrix system ensures that both active ingredients are slowly and continuously released from the collar towards the animal thereby avoiding peak concentrations and ensuring that acaricidal/insecticidal concentrations of both active ingredients are present in the cat's or dog's hair coat during the entire efficacy period. The active ingredients spread from the site of direct contact over the entire skin surface of the treated animal [8].

Following application of the collar, both active ingredients remain on the outer surface of the animal's skin and hair coat, enabling them to come into contact with the target parasites and display their efficacy. The neonicotinoid imidacloprid interacts with the nicotinic acetylcholine receptors (nAChRs) on the post-synaptic membrane [9], while flumethrin, as an α-cyano-(type II)-pyrethroid, exhibits excitatory efficacy by blocking the voltage gated axonic sodium channels [9]. As recently described in laboratory studies [9], imidacloprid and flumethrin have synergistic efficacy on insects, in particular fleas. Both active ingredients are well known on the ectoparasiticide market: imidacloprid has been the insecticidal active ingredient in products such as Advantage^®^, Advantix^® ^and Advocate^® ^since 1996 while flumethrin has been registered since 1986 for animal use and has already been used extensively in livestock animals (esp. cattle). It has also been used for more than a decade in the EU as an active ingredient in companion animal products (dogs and in parts of the EU also cats): Kiltix^® ^collar [6].

As usual for clinical field studies, a full set of laboratory efficacy and safety studies was a prerequisite for conducting the herein described studies. According to a set of preceding laboratory *in-vivo *studies the collar provides long term (8 month) prevention for cats and dogs against ticks (cats: *I. ricinus, R. turanicus (= the Rhipicephalus species on cats*, [1]*) *dogs: *I. ricinus, I. scapularis, R. sanguineus, D. reticulatus) *and fleas (*C. felis*,) [10,11]. The collar proved also to be effective against the tick species *Amblyomma americanum *(USA) and *Dermacentor variabilis *(USA) [10,11]. The collar's safety has been shown in longterm, overdosage studies following the guideline on target animal safety (VICH GL43) as well as US EPA requirements at up to 5 times the target dose in both cats and dogs, 10 week old kittens and 7 week old puppies [12]. Additionally, to this standard study set, a specific product safety concern for cats was addressed before study start: their particular sensitivity towards pyrethroids [13]. This sensitivity is generally explained by a reduced enzyme pattern for hydrolysis of pyrethroid-esters in cats, and by toxic metabolites developing during the pyrethroid degradation process combined with the reduced glucuronidation and therefore excretion capacity of the feline liver [14-16]. Opposite to e.g. permethrin and deltamethrin, the metabolism of flumethrin is simple without the need for glucuronidation. Flumethrin itself or its main metabolite flumethrin acid can be excreted without conjugation via feces [17]. Moreover, flumethrin acid is pharmacologically inactive [18]. The decreased feline glucuronidation rate is therefore toxicologically irrelevant [17]. Accordingly the NOAEL (No Observed Adverse Effect Level) for flumethrin is identical for dogs and cats [19]. The already rather low flumethrin toxicity in cats is complemented in this new product by two other aspects: only a very low hair coat concentration of this highly effective acaricide is necessary for high efficacy against ticks, and this low amount is released steadily from the collar without peak concentrations [9]. These three aspects together form the basis for the collar to be a useful application form for an effective and safe cat acaricide.

This results in a special benefit of the imidacloprid 10%/flumethrin 4.5% collar when tick and flea treatment is necessary in mixed cat and dog households. As the cat and dog products are identical, dogs living closely together with cats can be protected against ectoparasites without safety concerns about cats potentially ingesting critical amounts of active ingredient through mutual grooming.

Besides the classical target animal safety evaluation, the particular pharmaceutical application via a collar bears another safety relevant aspect for cats which was taken into account and tested before the collar was used in the discussed field study: in contrast to dogs, which usually wear constantly leather or chain collars, cats are less often equipped with these accessories by their owners. The reason for this is the different behaviour of this species; free roaming cats displaying the full range of their hunting instinct are known to roam through wood, scrub or other wayless terrain and to pass through particularly narrow apertures when hunting their prey. Cat owners often fear that cats may be captured or strangulated by a collar, which may get caught on any salient piece of wood or other rigid material, and so cats are perceived to be more at risk wearing a collar. The new collar has an integral safety-closure ratchet mechanism that is constructed to yield at approximately 50 Newton (~5 kg) traction [Jiritschka W, Imidacloprid/Flumethrin - collar: Determination of force needed to re-open the collar using the integral closing system, unpublished], and allows the collar to be widened by the distance to the next rib. Widening by two or three rib spaces will usually easily allow a cat to escape from the collar in the unlikely case that it gets trapped. 50 Newton is the force a 2 kg cat needs to jump to a height of just 40 cm. This is easily brought to bear by even a small cat, which seriously wants to escape from somewhere but is strong enough to keep the collar on the cat's neck under normal circumstances and to prevent the cat from accidentally losing it. Additionally the collar design prevents the collar from widening to the extent that can occur in certain elastic collars, where cats may be endangered by getting the front leg stuck in the over-widened collar; in this case severe skin damage may occur as the leg becomes hooked at the elbow and cannot be retracted by the animal itself.

A second, backup security feature for cats is represented by the "pre-determined breaking point" of the cat collar. In contrast to the approximate 140 Newton (~14 kg) traction necessary to break the cat collar itself, this pre-determined area breaks at approximately 80 Newton (~8 kg) [Jiritschka W, Imidacloprid/Flumethrin - collar: Determination of force needed to disrupt the collar, unpublished]. So even in cases where the safety-closure mechanism is blocked by any unfortunate accident, the animal has an increased chance to escape by breaking the collar.

The extended safety profile described above and initial efficacy data of the new collar in both cats and dogs was a prerequisite to apply for permission to use it in pets naturally infested with fleas and ticks under field conditions. The objective of the present therapeutic confirmatory, controlled, randomised, blocked, multi-centre and multi-regional field study was accordingly to confirm the long-term efficacy and safety of the combination of "imidacloprid 10%/flumethrin 4.5%" administered by collar for the treatment of natural infestations of fleas and/or ticks in cats and dogs, presenting as patients in European veterinary practices and based on statistical non-inferiority as compared with a licensed collar product for cats and dogs. Statistical non-inferiority of the imidacloprid/flumethrin collar (IVP) compared to the control product (CP) was shown if the lower limit of the two-sided 97.5% confidence interval of the difference between IVP and CP was greater than -15%.

## Methods

### Investigational Veterinary Product (IVP) and Control Product (CP)

The new imidacloprid 10%/flumethrin 4.5% collar under investigation (IVP) is a grey, odourless polymer matrix collar containing 10% (w/w) imidacloprid and 4.5% (w/w) flumethrin. The collar is designed to be fixed around the neck of cats and dogs and comes in two different sizes for dogs and one size for cats. The length of the collar can be adapted to the size of the animal with a ratchet closure mechanism and by cutting the overlapping end to the required length. Administration route, schedule, dose and dosage form of the IVP and the CP "Beaphar Ungezieferband fuer Hunde/fuer Katzen", a collar containing Dimpylat and authorised for dogs and cats for the control of flea and tick infestations in several EU countries including Germany are shown in Table 1.

**Table 1:** Products used and treatment regimen according to the product label requirements

ivp or CP	Route	Schedule	Dose (per animal)	Dosage Forms	Used for
Imidacloprid/Flumethrin collar (Seresto^®^)	Topical as a collar	Once on day 0	One collar, length adapted to size of animal based on neck size: 100 mg Imidacloprid and 45 mg Flumethrin/g collar	Small collar(≥ 1.0 kg to ≤ 8.0 kg bodyweight (b.w).) Large collar (> 8.0 kg b.w.)	Dogs/cats Dogs

Dimpylat collar (Ungezieferband fuer Hunde^®^)	Topical as a collar	Once on day 0 and day 140 ± 2	One collar, length adapted to size of animal based on neck size: Equal to 3.6 g Dimpylat/24 g collar	One collar size	Dogs

Dimpylat collar (Ungezieferband fuer Katzen^®^)	Topical as a collar	Once on day 0 and day 140 ± 2	One collar, length adapted to size of animal based on neck size: Equal to 2.1 g Dimpylat/14 g collar	One collar size	Cats

### General design

Two multicentre, multiregional positive controlled clinical field studies, one in cats and one in dogs, were conducted with the IVP imidacloprid 10%/flumethrin 4.5% collar, testing the safety and efficacy in tick and flea infestations in cats and dogs for 8 months and compared to a positive control product (CP) "Beaphar Ungezieferband fuer Hunde/fuer Katzen". These clinical field studies were carried out in 33 practices/clinics, and patients were actively enrolled from different geographical areas in Europe (France, Germany, Hungary and Portugal).

The IVP collar treatment was given once during the study except in cases when the animals lost the collar and it had to be replaced, whereas the CP with its shorter label efficacy of 5 months was replaced after 5 months of wear (approx. day 140). Non-inferiority of the IVP in comparison to the CP was tested comparing baseline to post-treatment parasite counts as observed on day 2 and then every 28 days post treatment.

### Procedure

After the informed owner consent had been obtained, animals were enrolled based on predefined in- and exclusion criteria. Depending on the identified parasites they were allocated either to the flea or to the tick subgroup of the total ***efficacy population (per-protocol population, PP)***. Within the subgroups, they were randomly allocated to treatment in a ratio of 2:1 to either the IVP or the CP group. For flea infestations (an animal with at least 5 viable fleas), the primary animal of a multipet household was the experimental unit and formed part of the efficacy population (per-protocol population). Other animals of this household were treated with either the same product as the primary animal (supplementary animals) or, if contraindicated, with a product of choice of the investigator (additional animals). For tick infestations, each individual animal hosting at least 3 viable and attached ticks was enrolled and the individual animal was the experimental unit. Primary and supplementary animals of the flea part of the study together with all tick patients formed the ***safety population (intention-to-treat population, ITT)***.

Day 0 was defined individually as the day an animal was found suitable for enrolment in the study and was treated with either treatment. Study completion was the day the animal completed the study, normally day 238, unless it was previously withdrawn from the study. Animals underwent parasitological and clinical assessments on day 0 and thereafter in monthly intervals (every 4 weeks) until day 238. These intervals were treated in the statistical evaluation as "post baseline periods". All visits (except visit on day 2: ± 1 day) were performed within a range of ± 2 days of the target day.

#### Parasitological examinations

Tick and flea counts were performed by the examining veterinarian as complete body counts according to a defined procedure, using manual palpation for ticks and additional fine-toothed flea combs for fleas. Counting was terminated at 10 minutes after the last identification of a flea or tick. Parasites were collected, fixed in ethanol and stored for species determination at a later stage. The species and developmental stage of the collected ticks from dogs and cats were identified by the Department of Comparative Tropical Veterinary Medicine, LMU Munich, Germany. The identification of collected fleas was performed by Bayer Animal Health GmbH, Monheim, Germany. Both institutes conducted the identification according to approved internal Standard Operation Procedures, using a number of morphological keys, either published (such as e.g. the Catalogue of the Rothschild Collection of Fleas [20]) or unpublished, internal expert's material (such as extensive descriptions and photos).

#### Clinical and safety observations

Primary animals were observed on day 2 and monthly thereafter for clinical signs, dermatological changes at the application site and adverse events, which were then categorized into suspected adverse drug reactions (SADR) and events unrelated to treatment. Supplementary animals were observed at least once at the end of the study by the study veterinarians for changes at the application site and for adverse events. Additionally, all animals were under daily supervision of their owners who were obliged to report any adverse events, especially signs of collar side effects, to their veterinarian as soon as they appeared.

### Data handling and analysis

Data from all study animals were entered into StudyBase^®^, an electronic data capture solution specifically designed for animal health studies via a web browser. Software was validated prior to use. After verification of the data, they were downloaded to SAS^® ^for analysis (SAS Institute Inc., Cary, NC, USA, version 9.2).

### Statistical analysis

The primary (overall) efficacy of the investigational veterinary product was defined as the average viable tick or flea count reduction compared to the day 0-baseline over the entire treatment period (8 months) and was compared to the control product using a test of non-inferiority corrected to baseline.

The percentage of flea and tick count reduction compared to baseline was calculated for each monthly evaluation period. Mean percentage reduction over all post-baseline periods was compared between treatment groups using a non-inferiority margin of 15%. Non-inferiority of IVP compared to CP was shown if the lower limit of the two-sided 97.5% confidence interval of the difference between IVP and CP was greater than -15%. This corresponds to the following test hypotheses:

$$\begin{array}{c} {\text{H}}_{0}:\text{P}{\text{R}}_{\text{IVP}}\leq \text{P}{\text{R}}_{\text{CP}}-15\%\\ {\text{H}}_{\text{A}}:\text{P}{\text{R}}_{\text{IVP}}>\text{P}{\text{R}}_{\text{CP}}-15\% \end{array}$$

with PR_IVP _resp PR_CP _being the least square means of percentage reduction over all post-baseline periods for the IVP compared to the CP calculated in an analysis of variance with repeated measurements adjusted for baseline (main effect of treatment over all post-baseline periods).

For calculation of the percentage parasite count reduction (PR) the following formula was used for each individual animal:

$$\frac{\text{count}\left(\text{period}\;1\right)-\text{count}\left(\text{period}\;\text{i}\right)}{\text{count}\left(\text{period}\;1\right)}\times 100=\text{PR}\;\text{at}\;\text{Period}\;\text{i}$$

for i = 2 to 11 (post-baseline evaluation periods) and period 1 = baseline.

As a secondary efficacy criterion, tick and flea count reductions (compared to baseline) were assessed separately for each single post-baseline period to evaluate superiority of the IVP compared to the control group.

Additionally the prevalence of concurrent tick and/or flea infestations was evaluated for the whole study period and the percentage of patients showing Flea Allergic Dermatitis (FAD) was calculated for each observation day.

## Results

The total ***safety population (intention to treat population, ITT) ***consisted of 400 dogs and 313 cats, of which 271 dogs and 232 cats were treated with the imidacloprid 10%/flumethrin 4.5% collar (IVP) and 129 dogs and 81 cats were treated with the CP (Table 2). The ***efficacy population (per protocol population, PP) ***consisted of 118 (IVP) + 47 (CP) cats and 197 (IVP) + 94 (CP) dogs. As common in this type of study, the number of control animals (CP group) was considerably smaller than the number of animals in the IVP group. The CP animal number was confirmed to be sufficient by the proof of statistical comparability of IVP and CP in terms of epidemiological data (gender, neutered, pure-bred, age, weight, coat length, husbandry and living place (urban or country side). All comparisons resulted positively in values between p > 0.05 up to 1.00, except one single value in the cat PP flea population (hair coat length; p = 0.048). The investigational product showed a long and reliable efficacy against fleas and ticks over the complete 8 months study period, which significantly exceeded the efficacy of the control product efficacy (except for tick efficacy in cats where superiority of the IVP was shown for months 2-4 only). A good tolerability of the IVP was shown with only a few (9% in cats/1.04% in dogs) minor local tolerance events mostly due to the mechanical influence of the collar, such as erythema, alopecia, scratching and cosmetic effects such as hair discoloration.

**Table 2:** Study design and allocation to treatment of safety population (= Intent to Treat Population, ITT) and *efficacy population (= Per Protocol Population, PP)*

Dog study			
**Group**	**Randomised as**		**Observation days**
		
	**tick patients**	**flea patients**	

"Imidacloprid and Flumethrin" (IVP)	139 *(114)*	132 *(83)*	Day 0
	
Dimpylat (CP)	63 *(52)*	66 *(42)*	Day 2 (+1)
	
Total evaluated	400 *(291)*		Day 28 (± 2)
			Day 56 (± 2)
			Day 84 (± 2)
			Day 112 (± 2)
			Day 140 (± 2)
			Day 168 (± 2)
			Day 196 (± 2)
			Day 224 (± 2)
			Day 238 (± 2)

**Cat Study**			

Imidacloprid/Flumethrin (IVP) Dimpylat (CP)	159 *(62)*	73 *(56)*	Day 0
		
	52 *(21)*	29 (27)	Day 2 (+1)
			Day 28 (± 2)
			Day 56 (± 2)
			Day 84 (± 2)
			Day 112 (± 2)
			Day 140 (± 2)
			Day 168 (± 2)
			Day 196 (± 2)
			Day 224 (± 2)
			Day 238 (± 2)

Total cats evaluated	313 *(166)*		

At enrolment, treatment groups were assessed for any differences regarding breeds, coat length, husbandry and tick and flea counts and no relevant differences were observed between groups. At enrolment (day 0), *C. felis *was found to be the most prevalent flea species identified in cats (96.8%) and dogs (94.0%), while *C. canis *(0% and 12.0%), *A. erinacei *(3.2% and 2.4%) and *P. irritans *(1.6 and 3.6%) were also identified. Tick species identified from viable ticks at day 0 in cats and dogs respectively were: *D. reticulatus *(1.8% and 18.4%), *I. hexagonus *(7.1% and 11.4%), *I. ricinus *(89.3% and 64.9%) and *Rhipicephalus spp. (*most probably *turanicus/sanguineus) *(8.9% and 28.1%). Other tick species identified in very low numbers included *Haemaphysalis *spp., *I. canisuga *and *Ixodes *spp. (Table 3).

**Table 3:** Tick and flea species identified on dogs and cats (as percentage of primary IVP animals) and percentage of patients with mixed flea and tick infestation

Results	Dogs	Cats
Flea species identified: (% of primary IVP animals)	*Ctenocephalides felis *(96.7%)	*Ctenocephalides felis *(91.9%)
	*Ctenocephalides canis *(0.5%)	*Ctenocephalides canis *(11.6%)
	*Pulex irritans *(0.5%)	*Pulex irritans *(2.0%)
	*Archaeopsylla erinacei *(3.3%)	*Archaeopsylla erinacei *(2.0%)

Tick species identified: (% of IVP animals)	*Ixodes ricinus *(89.2%)	*Ixodes ricinus *(64.9%)
	*Ixodes hexagonus *(8.8%)	*Ixodes hexagonus *(8.4%)
	*Rhipicephalus spec*. (8.8%)	*Rhipicephalus sanguineus *(28.2%)
	*Dermacentor reticulatus *(2.9%)	*Dermacentor reticulatus *(18.8%)

**Patients with mixed flea/tick infestations**	3.2% of animals	3.5% of animals
	5.3% of households	5.7% of households

### Efficacy

**Figure 1:**
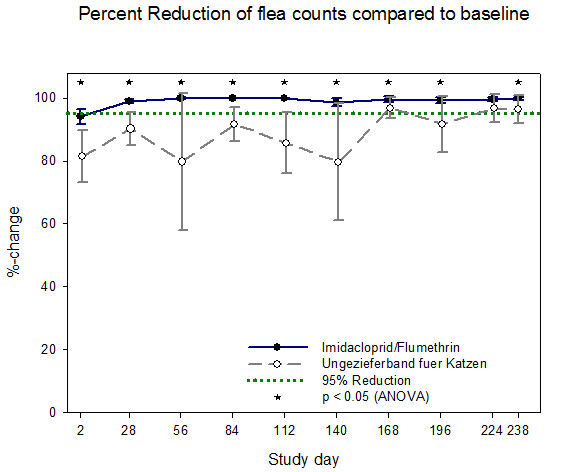
**Percentage reduction of viable fleas in cats for each efficacy evaluation period - arithmetic means and 95% confidence limits (PP population)**.

**Figure 2:**
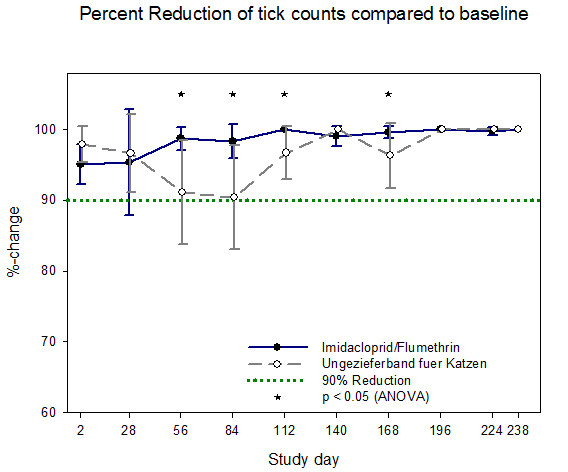
**Percentage reduction of viable ticks in cats for each efficacy evaluation period - arithmetic means and 95% confidence limits (PP population)**.

**Figure 3:**
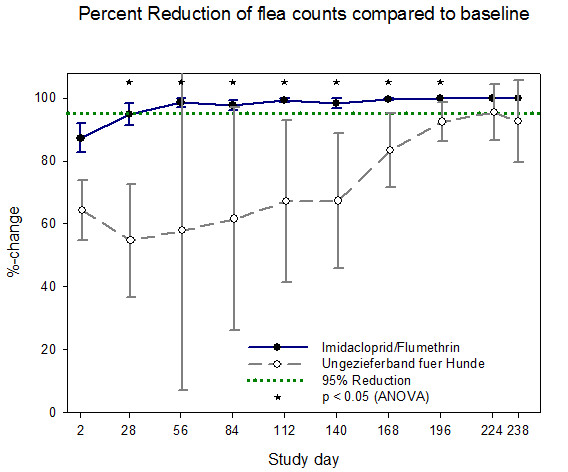
**Percentage reduction of viable fleas in dogs for each efficacy evaluation period - arithmetic means and 95% confidence limits (PP population)**.

**Figure 4:**
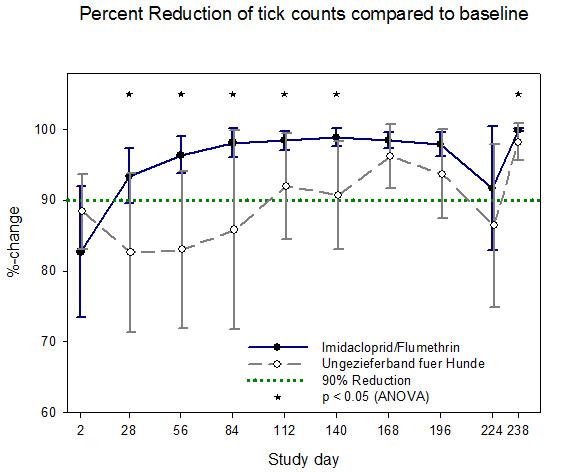
**Percentage reduction of viable ticks in dogs for each efficacy evaluation period - arithmetic means and 95% confidence limits (PP population)**.

Efficacy of the imidacloprid 10%/flumethrin 4.5% collar in comparison to the control product is illustrated for cats in Figures 1 and 2 and for dogs in Figures 3 and 4. All calculations are based on arithmetic mean data in accordance with the EU *Guideline for the testing and evaluation of the efficacy of antiparasitic substances for the treatment and prevention of tick and flea infestation in dogs and cats *(EMEA/CVMP/005/2000- Rev.2).

#### Overall efficacy against fleas and ticks (primary efficacy criterion)

Based on the ***efficacy population (per protocol population) ***the mean percent reduction of **flea **counts in the IVP group was 98.4% in cats and in 96.7% dogs, whereas in the CP group it was 79.3% in cats and 57.9% in dogs for the overall study period (Table 4). The mean percent reduction of **tick **counts in the IVP group was 98.4% in cats and 94.7% in dogs whereas in the CP group it was 96.9% in cats and 85.6% in dogs for the overall study period (Table 4). Non-inferiority (lower 97.5% confidence interval of least square mean difference greater than -15.0) was shown for the IVP groups (cats and dogs) compared to the control groups (cats and dogs). Based on the baseline counts of all animals treated ***(intention-to-treat population)***, superiority of the imidacloprid 10%/flumethrin 4.5% groups (cats and dogs) as compared to the control product was proven.

**Table 4:** Efficacy results based on tick and flea count reduction as compared to the baseline counts on Day 0

Results	Cat study				Dog study			
Efficacy **	**Fleas**		**Ticks**		**Fleas**		**Ticks**	

**Treatment group**	**IVP**	**CP**	**IVP**	**CP**	**IVP**	**CP**	**IVP**	**CP**

SD2-4 (96 h) curative	92.9*	71.8	94.8	97.7	86.7*	66.5	79.9	86.6

month 1	99.0*	87.1	94.0	96.3	94.1	48.6	93.4*	85.2

month 2	99.7*	57.1	99.3*	90.7	98.0*	28.2	97.8*	81.4

month 3	100*	90.2	97.9*	90.0	97.4*	48.6	98.8*	81.1

month 4	99.8*	76.9	100*	96.6	99.1*	56.1	98.2*	86.9

month 5	97.4*	68.1	99.4	100	97.5*	51.0	98.8*	83.4

month 6	99.1*	63.9	99.6	96.3	99.6*	60.5	98.5	88.0

month 7	99.8*	87.4	100	100	99.9*	67.3	97.9	88.0

month 8	99.3*	87.8	99.6	100	100	69.8	91.2*	79.9

m 8 (+14 d) (SD238)	99.8*	96.1	100	100	100*	67.8	99.8	90.7

Overall	98.3*	79.3	98.4*	96.9	96.7*	57.9	94.7*	85.6

*Efficacy of IVP is statistically superior to CP (p < 0.05; ANOVA adjusted for baseline or non-parametric Wilcoxon test), as evaluated from PP population**Efficacy calculations (PP population) were based on arithmetic means

#### Efficacy comparison per study day for ticks and fleas (secondary efficacy criterion)

##### Efficacy at day 2 (curative efficacy)

Curative efficacy against fleas and ticks already on the animal at the timepoint of treatment (day 0) is described by the study day 2 flea and tick count reduction. For fleas, it was 92.9% and 71.8% in cats and 86.7% and 66.5% in dogs for the IVP and CP group, respectively. For ticks, it was 94.8% and 97.7% in cats and 79.9% and 86.6% in dogs for the IVP and CP group, respectively.

##### Efficacy at day evaluation time points post day 2 (preventive efficacy)

From day 28 to day 238 the IVP group reached more than 95% percent **flea **count reduction based on both the cat (exception day 28 (94.1%)) and the dog efficacy population whereas the control group never reached the threshold of 95% flea count reduction during the 8 months study duration. The IVP was superior to the control product at every flea count time point in dogs and cats and at various tick assessment time points (Table 4). Superiority of the imidacloprid 10%/flumethrin 4.5% collar was tested using ANOVA adjusted to baseline at p < 0.05. From day 28 to day 238 the IVP group reached more than 90% percent **tick **count reduction based on both the cat and the dog efficacy population whereas the control group showed values above 90% only in cats and did not reach at any time within the 8 months 90% tick count reduction in dogs.

The non-parametric Wilcoxon test confirmed the results of the ANOVA analysis.

#### Concurrent flea and tick infestations

Concurrent flea and tick infestations were observed in 3.2% and 3.5% of the dogs and cats and in 5.3% and 5.7% of the assessed households (Table 3).

#### Flea Allergic Dermatitis (FAD)

The influence of IVP and CP treatment on FAD was evaluated for both treatment groups. In addition, to evaluate the curative efficacy of the new collar in particular, and in order not to overestimate its efficacy on FAD, the individual clinical case records of the IVP group were further investigated to evaluate the influence of palliative glucocorticoid treatments to the treatment success:

##### Cats

On the enrolment day (study day 0), 18 (7.8%) of the 231 cats in the IVP-ITT population showed symptoms of FAD. By SD 2 this number was reduced to 14 (6.1%) and FAD was completely cured at the SD 28 evaluation. Only one new case came up at SD 196. Since no further investigations were conducted in this animal, the reason/correct diagnosis cannot be definitively clarified, especially since the flea counts were zero. In the CP group, 8 (10%) out of the 80 cats in the ITT population started the study with FAD already present. FAD cases in the CP group also declined rapidly within the first 28 days after treatment to between very few to no flea allergic animals.

##### Description of palliative FAD treatment in the IVP group

Palliative treatment was not prohibited for the study animals. Only five FAD positive cats were concomitantly treated once with a glucocorticoid against FAD at SD0 in parallel to the collar application, and only one of these treatments contained a depot formulation. Of the 18 FAD cases found in the cat population, the 4 short term and one long term glucocorticoid treated cats represent 28% of this subgroup and the rest of the cases resolved without any additional treatment.

##### Dogs

On the inclusion day, 23 (8%) of the 286 dogs in the ITT population showed symptoms of FAD. By SD 2 this number was reduced to 16 (5.6%), and consequently decreased to 4 (1.4%) at SD 28. FAD completely vanished at the SD 56 evaluation, apart from one persistent case, which remained visible up to SD 168 before a full cure was achieved. At the very end of the study (SD 238), two new cases came up. Since no further investigations were conducted in these animals, the reason/correct diagnosis cannot be definitively clarified, especially since the flea counts were zero. In the CP group, 12 (8.8%) out of the 136 dogs in the ITT population started the study with FAD already present. FAD cases in the CP groups also declined rapidly within the first 28 days after treatment to between very few to no flea allergic animals.

##### Description of palliative FAD treatment in the IVP group

Only one FAD positive dog was concomitantly treated once with a short term glucocorticoid against FAD at SD0 in parallel to the collar application, moreover, one FAD positive dog at study inclusion had a history of being treated one month before collar application with a glucocorticoid. Of the 23 FAD cases found in the dog population, the one short term glucocorticoid treated animal represents 4.3% of this subgroup whereas the rest of the cases resolved without any additional treatment.

### Safety

The number of adverse events suspected to be treatment related was evaluated for both treatments and evidence was compared statistically. In cats, a total of 28 events were suspected to be related to study medication (Suspected Adverse Drug Reactions (SADRs)), 23 in the IVP (9.0% of the IVP safety population) and 5 in the control group (5.6% of the CP safety population). This difference was not statistically significant (p > 0.4; Fisher's exact test). Cat SADRs were generally mild dermal reactions (alopecia, pruritus, mild contact dermatitis).

In dogs, in the IVP group 3 events (alopecia, hair coloration, dermatitis) and in the CP group 4 events (alopecia, flea infestation, pruritus, aggressive behaviour towards a collar wearing animal) were scored as being related to the study medication. The difference between the two groups was not statistically significant (P > 0.16; Fisher's exact test)

## Discussion

### General

The two studies reported here were conducted according to VICH GCP, which assured the accurateness, integrity and correctness of the observations. The studies were controlled by a positive control group, animals were randomised to treatment groups and the laboratory scientists conducting the identification of parasites were blinded with respect to treatment group. Although the investigators counting the fleas and/or ticks could not be blinded due to the nature of the treatments, the possible influence of bias is nevertheless limited: parasite counts are an objective measure and a standard procedure was followed to guarantee accurate and comparable parasite counts at all study sites throughout the study period.

### Efficacy

The evaluated flea counts confirm the results described for imidacloprid and imidacloprid combinations by various authors [21-24]. However, the duration of efficacy against fleas of the previously licensed spot-on formulations of imidacloprid is about one month for Advantage^®^, Advocate^® ^and Advantix^® ^(Bayer Animal Health) respectively, while the collar exhibits a long-term efficacy for 8 months. The same applies to ticks, where the efficacy duration of spot on products is usually limited to 3-4 weeks, depending on tick species (Advantix^®^, Frontline Combo^®^) or, for cats is even shorter (2 weeks; Frontline Combo^® ^cat). The therapeutic need in cats is quite evident as, apart from this product, the availability of acaricides for this species is highly limited.

Efficacy for a period of eight months had been proven as prerequisite for the field study in fleas and numerous tick species in laboratory studies reported by Stanneck *et al. *[10,11]. The fast onset of protective insecticidal, acaricidal and repellent efficacy (immediately in fleas, as to 24 h counts; within 48 hours in ticks) together with the reliable longterm insecticidal, acaricidal and repellent efficacy in the various European flea and tick species became evident in the course of the described laboratory work and has been confirmed now by the excellent results of the herein described field studies. This longterm efficacy is useful for full season protection in most climatic areas, where fleas and ticks exhibit a significant problem. Overgaauw [25] reports that the main problem in the control of flea and tick infestation is caused by the inadequate frequency and duration of treatments given by owners. According to Overgaauw, 62% of treatment failures are caused by inadequate treatment frequency and duration. The imidacloprid 10%/flumethrin 4.5% collar offers unique options for the easy, long-term and sustained control of fleas and ticks and may help overcome the problems of owner compliance.

### Flea allergic dermatitis

The one group of animals that is highly dependent on strict owner compliance especially for flea control are those suffering from FAD. Although any modern flea protective spot on product states on its label that treatment aids FAD control, FAD patients prescribed these products frequently suffer from the intermittent in- and decrease of flea populations due to inadvertently prolonged inter-treatment intervals caused by poor owner compliance. The imidacloprid 10%/flumethrin 4.5% collar showed in the field studies a high potential to cure and prevent FAD: out of 18 (cat) and 23 (dog) FAD cases found in the cat and dog population the 5 (cat)/1 (dog) initial glucocorticoid treated animals represent 28% (cat) and 4.3% (dog) of this subgroup whereas the rest of the cases (72% (cat) and 95.7% (dog)) resolved without any additional treatment. The FAD curative efficacy of the collar is therefore obvious, as is the protective efficacy: no further glucocorticoid treatment was necessary in this highly flea susceptible subpopulation throughout the study to prevent further FAD relapses.

Moreover, in one FAD positive dog which was treated one month before the start of the study with a glucocorticoid, obviously with limited success (FAD present at study enrolment) the FAD resolved under exclusive treatment with the imidacloprid 10%/flumethrin 4.5% collar without any further palliative treatments. The benefit of FAD treatment by the strict and sustainable flea eradication coupled with longterm continuous flea control over just palliative measures is evident.

### Safety

With the exception of local reactions at the collar site, no adverse event in either dogs or cats was evaluated as being product related. Mainly, the reported cases were slight signs of dermal irritations most probably due to mechanical rubbing as they all had one thing in common: they were generally transient and healed under the collar when the collar was left on the animal. These observations reflect the experiences made in the extensive series of up to 5 times overdosed longterm laboratory target animal safety studies in cats and dogs, kittens and puppies which were conducted as a prerequisite for conducting the field studies [12]. These studies showed that the formulation is safe for cats (from 10 weeks of age) and dogs (from 7 weeks of age) even at a 5 fold overdose and with repeated (every 2 months) applications. Particularly important was the confirmation of collar safety in the more drug sensitive species i.e. cat, especially as the collar contains flumethrin, a member of the pyrethroid class. As described in the introduction, flumethrin as an active ingredient was found and proved to be as safe in cats as in dogs. Additionally, the active ingredients are released from the collar matrix evenly and without peak concentrations [9] so that no elevated acute active ingredient exposure occurs. Having safe active ingredients in a safe, slow release formulation makes the collar an ideal application for the drug sensitive cat species and allows the use of flumethrin, a potent acaricide with fast acting, repellent properties, in a species in which it is not possible to apply any of the current pyrethroids (e.g. permethrin, deltamethrin). The undeniable safety of the collar particularly for cats was additionally underlined by its mechanical features, which were especially designed to address the particular concerns of cat owners. Cat owners may be worried about fitting collars to cats as they perceive an increased potential risk for cats to be caught or strangulated while roaming and hunting outside. The specific safety oriented design (safety closure mechanism plus a predetermined breakpoint as second fall back safety feature) will release a cat with comparably minor extra force. This is reflected by the results of the cat field study in which no such events were recorded.

### Canine vector borne diseases (CVBD)

The above described efficacy gets medical importance when it is seen in the light of the role of ectoparasites as potent vectors of bacterial, viral and protozoal diseases, as briefly outlined in the background section. Besides offering protection against the parasites themselves and the establishment of large populations (fleas, *R. sanguineus *ticks), an uninterrupted longterm efficacy has another important benefit; by prevention of parasite bites it also can help preventing vector borne disease transmission. This is obvious in regions with regular and high flea and especially tick infestation pressure but holds also true for regions with more occasionally occurring flea and tick infestations in which animal owners tend to rely on their own abilities in finding and removing attached ticks by hand quick enough to prevent pathogens being transmitted. This is a dangerous misinterpretation of the physiological facts. On the one hand, there is a highly underestimated parasite species which can act as fast and potent transmitters of vector borne diseases: the fleas. Besides the well known and by Europe's pet owners no longer feared plague bacteria, they carry a number of e.g. *Rickettsia *or *Bartonella *species which are in many cases zoonoses and therefore of remarkable medical impact. Especially Bartonellosis, caused by *B. henselae *and mainly linked to cats as a reservoir, can be regarded as one of the major potential emerging infections of man [26].

On the other hand even not all tick borne pathogens are transmitted as slowly as e.g. *Babesia canis canis *with the sporozoites needing a maturation of at least 48 hours in the tick's salivary glands to become infectious [27]. There are numerous diseases which are transmitted much faster (e.g. *Ehrlichia canis *within 4-6 hours after tick attachment) [28] and a small, attached but still unengorged female tick can be easily overlooked and so successfully transmit diseases during this early stage.

The danger of acquiring a tick borne disease is even increased by the fact that not only the easily visible adult ticks but also tick larvae and nymphs are highly important for CVBD transmission according to recent knowledge [29]. These parasites are nearly invisible because of their very small size and would not be noted or removed by the animal owner, but they are potent vectors due to horizontal transmission (larvae are infected and transfer the pathogen during moulting to the nymph stage) or even vertical transmission (an infected adult transfers the pathogen via the eggs to the next larvae/nymph generation: occurs e.g. in *Borrelia, Babesia*) [30]. As the number of the juvenile stages usually exceeds by far the number of adult ticks, they form a serious threat for animals exposed to their habitats. The susceptibility of tick stages against imidacloprid/flumethrin declines from larva > nymph > adult ticks. According to the European guideline EMEA/CVMP/005/2000, this is expected, but it was furthermore proven in particular during the development of the imidacloprid 10%/flumethrin 4.5% collar [9].

## Conclusion

Seresto^®^, an Imidacloprid 10%/Flumethrin 4.5% collar has been shown to be safe and highly efficacious in the treatment and prevention of tick and flea infestations in cats and dogs treated as patients presenting to veterinary practices under field conditions. The evaluation of efficacy was based on non-inferiority to the control group treated with a commercial product licensed for this indication. Efficacy for both tick and flea infestations was confirmed by at least 90% tick and 95% flea count reduction in cats and dogs, respectively for the whole study period. In addition, superiority of the IVP group compared to the control group was confirmed for both flea and tick count reduction at various time points.

Containing two highly potent yet safe active ingredients, the neonictinid imidacloprid and the α-cyano-pyrethroid flumethrin, the imidacloprid 10%/flumethrin 4.5% collar with its slow release formulation proved to be very safe in both dogs and cats. Especially for cats, this product provides the first longterm acaricidal and tick repellent treatment on the market.

The imidacloprid 10%/flumethrin 4.5% collar will thereby contribute markedly to the effective and safe protection of cats and dogs against ectoparasites and consequently vector borne diseases.

## Competing interests

This clinical study was completely funded by Bayer Animal Health GmbH, Monheim, Germany, thereof D. Stanneck (Germany) and C. LeSueur (France) are employees. KLIFOVET AG is an independent Contract Development Organisation, which was contracted to manage the conduct of this study. K. Hellmann is the managing director, I. Radeloff is the project manager, J. Rass an employee. All authors voluntarily publish this article and have no personal interest in this trial other than publishing the scientific findings that they have been involved in by planning, setting-up, monitoring, conducting and analysing this study.

## Authors' contributions

DS, JR, IR, CLS and KH designed the study design and protocols and JR, IR and CLS carried out the studies. DS, JR, IR, CLS and KH compiled and analysed the data and were contributing substantially to the final study reports. JR, IR and KH were responsible for the first setup up of the manuscript, which was then substantially revised by all authors. All authors read and approved the final manuscript.

## Compliance statement

This study was conducted in France, Germany, Hungary and Portugal after obtaining the express authorisation of the relevant regulatory authority of these countries. Prior to treatment of any animal an informed owner consent was obtained in writing.
